# Rebalancing SMAD7/SMAD3 Signaling Reduces Adhesion Formation during Flexor Tendon Healing

**DOI:** 10.4014/jmb.2209.09033

**Published:** 2023-02-15

**Authors:** Ke Jiang, Yuling Li, Chao Xiang, Yan Xiong, Jiameng Jia

**Affiliations:** 1 Department of Orthopaedics, Affiliated Hospital of North Sichuan Medical College, Nanchong, P.R. China; 2 Key Laboratory of Emergency and Trauma, Ministry of Education, College of Emergency and Trauma, Hainan Medical University, Haikou, P.R. China; 3 Department of Orthopaedics, Daping Hospital, Army Medical University, Chongqing, P.R. China; 4 Department of Rehabilitation, Affiliated Hospital of North Sichuan Medical College, Nanchong, P.R. China

**Keywords:** smad7, smad3, adhesion formation, flexor tendon healing, mouse, injury

## Abstract

Transforming growth factor-β is a key factor in regulating adhesion formation during tendon healing. We investigated the effectiveness of SMAD family members, SMAD7 and SMAD3, in the TGF-β/Smad signaling during flexor tendon repair. Mouse flexor toe deep tendon rupture anastomosis models were made. On days 3, 7, 14, 21, and 28, the expressions of smad7 and smad3 in flexor tendon tissues were detected by RT-qPCR and western blot. Furthermore, postoperative intraperitoneal injections of SMAD7 agonists or SMAD3 antagonists were given. The degree of tendon healing was evaluated by adhesion testing and biomechanical experiments. Hematoxylin and eosin (HE) staining was used to observe the pathological changes. Immunohistochemistry was used to evaluate the expressions of collagen III, SMAD3, and SMAD7. The mRNA levels of matrix metalloproteinases, *Mmp2* and *Mmp9*, and scleraxis (SCX) in flexor tendon tissue were detected by RT-qPCR. *Smad3* expression increased and *Smad7* expression decreased in flexor tendon tissue after injury. In addition, the SMAD7 agonist blocked SMAD3 phosphorylation. SMAD7 agonist and SMAD3 antagonist both improved adhesion formation during flexor tendon healing, and decreased the expressions of collagen III, *Mmp9*, and SCX, while increasing *Mmp2* expression. This study provides a possible theoretical basis for the SMAD7-SMAD3 signal cascade during flexor tendon adhesion healing.

## Introduction

Flexor tendon injury is a common trauma in hand surgery. It is difficult to repair because of the complexity of the tendon structure. Tendon adhesion is the most common complication, and its incidence rate is 2%-27%[1]. Tendon adhesion leads to poor recovery, and patients often need stage II tenolysis, which increases the pain and burden of the patients [2]. With the continuous improvement of microsurgical suture methods and the further improvement of rehabilitation programs, the results of tendon repair surgery have been greatly improved. However, peritendinous adhesion after suturing causes the tendon to lose its normal sliding performance and ultimately leads to the unsatisfactory recovery effect of hand function, which is still a major problem to be solved urgently in the field of hand surgery [3].

Tendon healing includes two processes, namely endogenous and exogenous healing, in which excessive exogenous healing is the main cause of tendon adhesion. During exogenous healing, fibroblasts grow toward tendon injury [4]. Meanwhile, local inflammatory exudation and proliferation, and healing of the tendon itself are the main causes of tendon adhesion [5]. After a tendon injury, synovial cells of the tendon sheath are damaged and secondary inflammation occurs [6]. Macrophages and neutrophils release a variety of cytokines to stimulate the proliferation of peripheral fibroblasts, and then inhibit the growth of tendon cells and matrix synthesis, leading to irreversible apoptosis of synoviocytes [7]. The process of restoring tendon fiber strength to promote tendon healing can lead to the adhesion of fibrotic scar tissue, resulting in the loss of postoperative tendon slippage and finger dysfunction [8].

At present, minimally invasive surgery, biological materials, and early postoperative function exercise are used to minimize the damage to the tendon, but still not entirely satisfactory [9]. Since a series of research achievements in recent years have preliminarily clarified the signal transduction mechanism of the transforming growth factor-β (TGF-β) superfamily in the treatment of tendon adhesions [10], and the intermediary molecule Smads serves as the main intracellular transduction molecule of signal from receptor to nucleus, whose role in the formation of fibrotic lesions such as hypertrophic scar has attracted the attention of many scholars [11, 12]. SMAD3 and SMAD7 are key factors in the TGF-β/SMAD signaling pathway, TGF-β realizes their functions through the Smad family [13]. Studies have shown that Smads exert bidirectional regulation on fibroblasts during the formation of fibrotic lesions such as hypertrophic scar. SMAD3 is the bridge between the membrane receptor and target gene, and SMAD7 can prevent SMAD3 from forming complex and SMAD3 phosphorylation [14]. These two proteins are the main inhibitory regulatory proteins in the TGF-β1 transduction pathway [15]. In addition, the activation of SMAD3 is a key link in fibrotic lesions, which mediates the biological effects of TGF-β and participates in the regulation of fibroblast proliferation, differentiation, inflammatory response, and collagen synthesis. Therefore, it plays an important role in wound healing, scar formation, and tissue remodeling [16]. Other studies have also shown that an imbalance in the *Smad7*/*Smad3* cascade leads to other phenotypic abnormalities, such as epithelial mesenchymal transformation [13, 17].

We investigated the possible regulatory mechanism of SMAD3 and SMAD7 proteins in non-scar tendon healing in vitro before [18]. Here, studying them in vivo may be an effective treatment against adhesion formation.

## Materials and Methods

### Murine Flexor Tendon Healing Model

A total of 78 female BALB/c mice (aged 6-8 weeks, 25 ± 2 g) were adaptively fed for 1 week. Pentobarbital sodium (Sigma Aldrich; CAS: 57-33-0) was used to induce mice anesthesia. After routine disinfection, the Mice proceeded to deep flexor toe tendon rupture and anastomosis. Procedure is as follows. All mice were dissected laterally in the middle toe of the hind paw to expose the flexor digitorum tendon. The flexor tendon was then cut laterally. After wounds were sutured, antibiotics were injected from day 1 to day 3 after surgery to prevent infection. Performed two separate experiments in subsequent studies. In the first experiment (I): 30 mice were grouped according to the time of tendon repair, time points as days of 3, 7, 14, 21, and 28 (*n* = 6) during flexor tendon healing. The mice with any drug treatment. In the second experiment (II): 48 mice were randomly divided into two subgroups. SMAD3 antagonist (10 mg/kg) (*n* = 24) or SMAD7 agonist (10 mg/kg) (*n* = 24) was injected intraperitoneally after surgery every 3 days until day 14 (*n* = 12) or day 21 (*n* = 12). The control (day 14 or day 21) group received equal saline.

### Adhesion Testing and Gliding Resistance

Adhesion testing was performed on post-repair day 14 and day 21 after repair. After the mice were sacrificed, the hind limbs were separated from the knee joint and each flexor digitorum longus (FDL) tendon was released from the tissue surrounding the proximal tarsal tunnel. The flexor digitorum tendon was secured with two straps and the limb was secured to a custom-made device that tightly clamped the tibia to prevent rotation. The inspector straightened the toes, allowing them to return to the vacancy, and then performed digital imaging to determine the neutral position of the metatarsal-toe joint (zero loads). An incremental load (0-19 g) was then applied to the FDL muscle. Digital images were used to quantify the flexion angle of the metatarsal phalanx relative to the neutral position and the corresponding loads were graphed. Sliding resistance was determined by a single-phase exponential equation fitting the bending data in a previous manner[19, 20], Among this, metatarsophalangeal (MTP) bending angle = β × (1-exp(-m/α), M was regarded as the applied load (Prism GraphPad 6.0A; GraphPad Software, Inc., USA). Adjusted the curve fitting by the maximum buckling angle (β). The load was previously determined to be 19 g, 75° for normal tendon applications [21]. Determined the sliding resistance (α) by nonlinear regression [22, 23]. The difference in buckling Angle between 0 g and 19 g loads was determined as the MTP range of buckling motion (ROM).

### Biomechanical Testing

We assessed the changes in the flexor tendon biomechanical properties at 14 and 21 days by the 8841 Instron DynaMight Axial Servo-hydraulic Test system (Instron Industrial Products, USA). Cut the middle toe flexor deep toe tendon of the posterior claw at the metatarsophalangeal joint approximately 1.5 cm in length, and then the kirschner wire was used to cut through the proximal 1/3 and distal 1/3 lengths of the finger shaft. The Kirschner wire was tightened on the dorsal side of the phalanx shaft. Then secure the Kirschner wire to the dorsal side of the phalanx with a clamp. In addition, we wrapped the deep flexor digitorum tendon in heavy sandpaper and attached it directly to the upper clamp to make sure to keep the tendon in line. Finally, until failure, carried out the tension displacement control test at a 30 mm/min speed. automatically recorded and plotted the Force-displacement data to determine the maximum load.

### Hematoxylin and Eosin (H&E) Staining and Masson Staining

Harvested the whole hind limbs at 14 and 21 days after post-repair [24] fixed them in 10% neutral buffered formalin for 48 h and washed in phosphate-buffered saline (PBS), then decalcified in 14% EDTA (pH 7.2) for 14 days at room temperature. The decalcified tissues were dehydrated with gradient ethanol, subjected to xylene transparency, dipped in wax, embedded in paraffin, and cut into 5 μm slices. Finally, H&E (Beyotime, China) and Masson staining kit (Beyotime) were used for sections staining by the manufacturer's instructions. Finally, they were dried and coated in neutral resin, followed by light microscopy. ImageJ image analysis software was used for analyzing the area of fibrosis (%) in samples.

### Immunohistochemical Staining

Incubated with 3% hydrogen peroxide after deparaffinized and rehydrated. Then, blocked the Tendon tissues samples with 3% BSA (Beijing Solarbio Science & Technology Co., Ltd., China) at room temperature and incubated overnight at a constant temperature of 4°C with the following antibodies: COLLAGEN III (ab7778; Abcam; dilution 1:100, UK); SMAD3 (ab40854; Abcam; dilution 1:1000); SMAD7 (ab216428; Abcam; dilution 1:500). The sections were washed three times with PBS, and treated with secondary antibody (1:2000, cat. no. ab205718; Abcam) for 20 min at 37°C. Finally, diaminobenzidine (DAB) staining was performed. Observed the positive expression area and strength of a 100× optical microscope. Two pathologists, unaware of treatment groups, evaluated all sections.

### RT-qPCR

The flexor tendon tissues (3, 7, 14, 21, and 28 days after repair, and 14 and 21 days after repair in the SMAD3 antagonist group and SMAD7 agonist group) were excised and frozen with liquid nitrogen. Total RNA was extracted using TRIzol reagent (Takara, China). Then, cDNA was synthesized using Prime Script RT Master Mix (Takara) with the primers (Table 1). The cDNA was amplified using the SYBR real-time polymerase chain reaction (PCR) master mix (Toyobo, Japan) kit according to the manufacturer’s instructions. Amplification was performed using the ABI 7000 Taqman system (Applied Biosystems, USA). The relative expression of *Mmp2*, *Mmp9*, *Smad3*, *Smad7*, and *Scx* was quantified using the 2^-ΔΔCt^ method, and *β-actin* was the internal control. The specificity of the PCR reaction was determined by the melting curve.

### Western Blot

The flexor tendon tissues (3, 7, 14, 21, and 28 days after repair in the flexor tendon injury healing models, and 14 and 21 days after repair treated with SMAD7 agonist models) from mice were frozen in liquid nitrogen and lysed in radioimmunoprecipitation assay (RIPA) lysis solution supplemented with 2 mM phenyl methylsulfonyl fluoride (PMSF). Extracted the protein by extraction kit (Pierce, Thermo Fisher, Ltd., USA). determined the protein concentration by BCA kit. A total of 30 μg protein was run per lane of 10% sodium dodecyl sulfate polyacrylamide gel electrophoresis (SDS-PAGE) gel. Transferred Gels to polyvinylidene fluoride (PVDF) membranes (Thermo Fisher Scientific, Inc., USA) after overnight incubation with primary antibodies specific for SMAD3 (1:1000, ab40854; Abcam), p-SMAD3 (1:2000, ab52903; Abcam) and SMAD7 (1:500, ab216428; Abcam) at 4°C. Incubated membranes for 1 h at room temperature with horseradish peroxidase (HRP) conjugated secondary antibody (1:2000, cat. no. ab205718; Abcam). After X film exposure and development, used Bio-Rad automatic gel imaging system for imaging preservation. Antibodies to β-actin (1:5000, ab8227; Abcam) were applied to provide an internal reference.

### Statistical Analysis

SPSS 20.0 (SPSS Inc.; USA) software was used for all statistical analyses, and Graph Pad Prism 6.0 software was used for drawing. All dates are expressed as the mean ± standard deviation (SD). Results were analyzed using one-way ANOVA and chi-square tests. *p* < 0.05 was considered statistically significant.

## Results

### *Smad3* Expression Increased and SMAD7 Expression Decreased in Flexor Tendon Tissues after the Injury

**Fig. 1:**
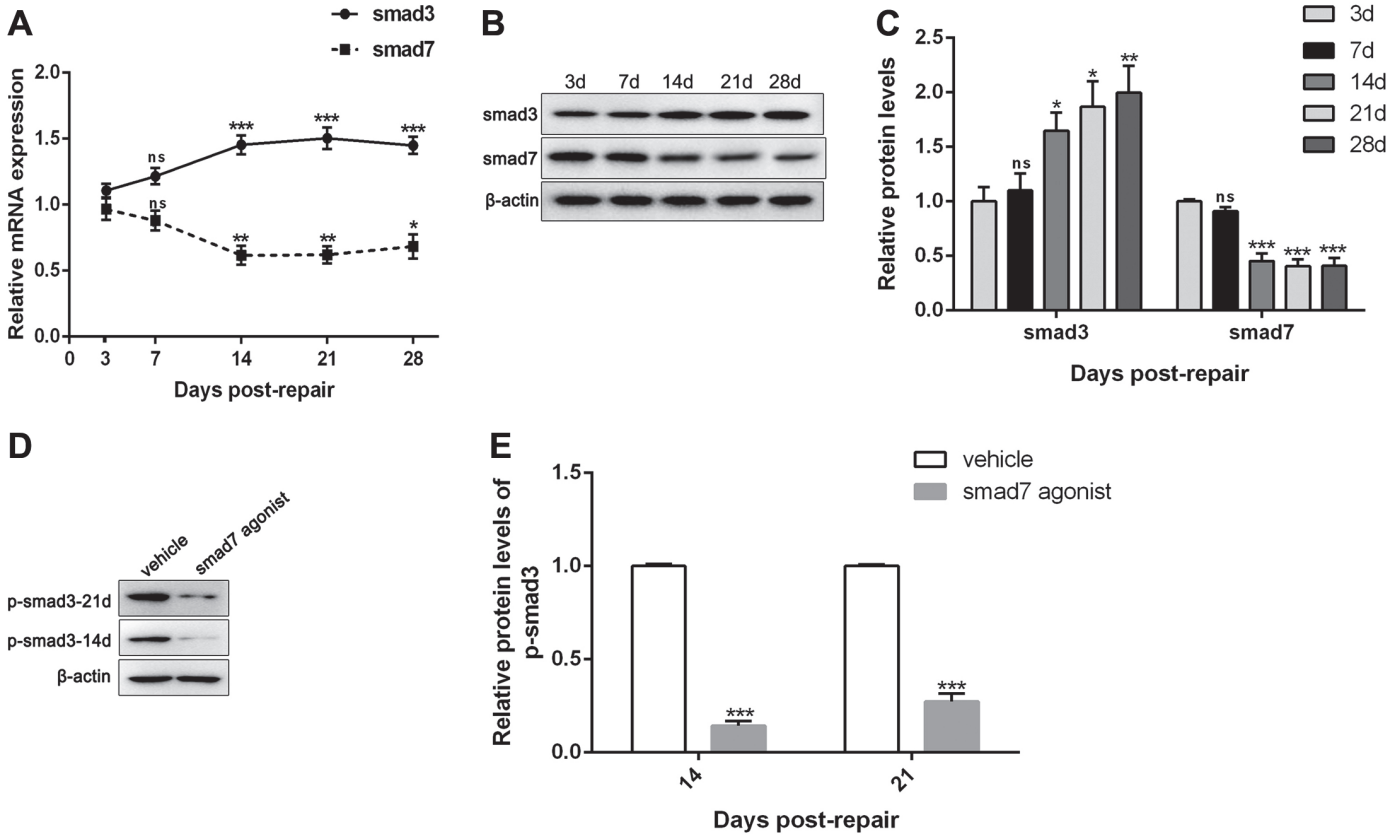
The expression of *Smad3* and *Smad7* in flexor tendon tissue of post-injury healing models. (**A**) Relative mRNA expressions of *Smad3* and *Smad7* in FDL tendon tissues by RT-qPCR at day 3, 7, 14, 21, and 28 post-repair. ns, vs. day 3; **p < 0.01, ***p < 0.001, vs. day 7. (**B**) Protein bands for smad3 and smad7 by western blotting assay at days 3, 7, 14, 21, and 28 postrepair. (**C**) Relative protein expressions of SMAD3 and SMAD7 in FDL tendon tissues by western blotting assay at day 3, 7, 14, 21, and 28 post-repair. Expression was normalized to β-actin. ns, vs. day 3; *p < 0.05, **p < 0.01, ***p < 0.001, vs. day 7. (**D**) Protein bands for SMAD3 by western blotting assay at days 14 and 21 post-repair. (**E**) Relative protein expressions of SMAD3 in FDL tendon tissues by western blotting assay at day 14 and 21 post-repair. Expression was normalized to β-actin. ***p < 0.001, vs. vehicle.

To determine the expression profile of *Smad3* and *Smad7*, a model of FDL tendon repair during the transverse phase of the muscle was used in untreated mice, which would inform the treatment timing for SMAD3 antagonist and SMAD7 agonist in the groups. Expression of *Smad3* mRNA continuously rose from day 3 to day 14 and peaked at day 14 post-repair. Besides, the levels of *Smad3* mRNA expression were significantly elevated and then largely maintained from day 14 to day 28 day. In addition, the expression of *Smad7* mRNA was shown to be significantly reduced in a continuous process from day 3 to day 14 and bottomed at day 14 post-repair, and they were then maintained until day 28 (Fig. 1A). Furthermore, similar results were further detected by western blot assay (Figs. 1B and 1C).

### SMAD7 Agonist Blocked SMAD3 Phosphorylation

To further confirm the relationship between SMAD3 and SMAD7, a systemic SMAD7 agonist or its vehicle control was given by intraperitoneal injection on days 3 to 8 after surgery. The results showed that the SMAD7 agonist significantly reduced the expression of SMAD3 phosphorylated protein (Figs. 1D and 1E).

### SMAD3 Antagonist and SMAD7 Agonist both Increased MTP Flexion Angle and Decreased Sliding Resistance

**Fig. 2:**
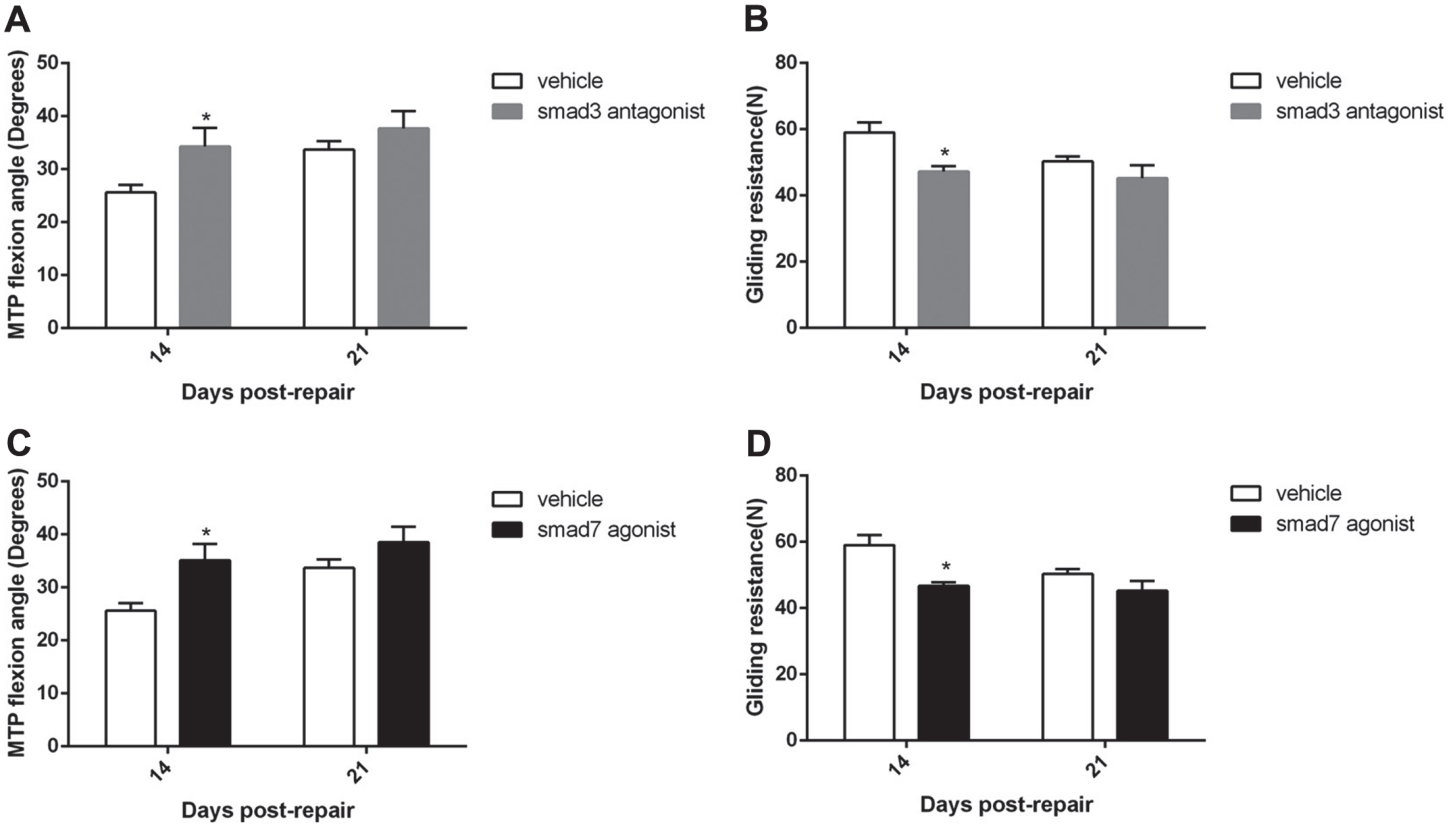
During flexor tendon healing, SMAD3 antagonist and SMAD7 agonist increased MTP flexion angle and decreased sliding resistance significantly. (**A, B**) MTP flexion angle was increased (**A**) and gliding resistance was decreased (**B**) by systemic SMAD3 antagonist during flexor tendon healing. *p < 0.05, vs. vehicle group. (**C, D**) MTP flexion angle was increased (**C**) and gliding resistance was decreased (**D**) by systemic SMAD7 agonist during flexor tendon healing. *p < 0.05, vs. vehicle group.

To investigate the effect of SMAD3 antagonist and SMAD7 agonist on tendon adhesion healing, we preliminary used adhesion testing to detect the metatarsophalangeal and gliding resistance of flexor tendon healing. The MTP flexion angle was significantly increased and the sliding resistance was significantly decreased in the SMAD3 antagonist group on day 14 post-repair compared to their corresponding vehicle group. On day 21 post-repair, the MTP flexion angle and gliding resistance were of no significance in the SMAD3 antagonist group compared with their respective vehicle groups (Figs. 2A and 2B). Moreover, the SMAD7 agonist played a similar role to the e SMAD3 antagonist (Figs. 2B and 2D).

### SMAD3 Antagonism and SMAD7 Agonism Changed Mechanical Strength and Stiffness

**Fig. 3:**
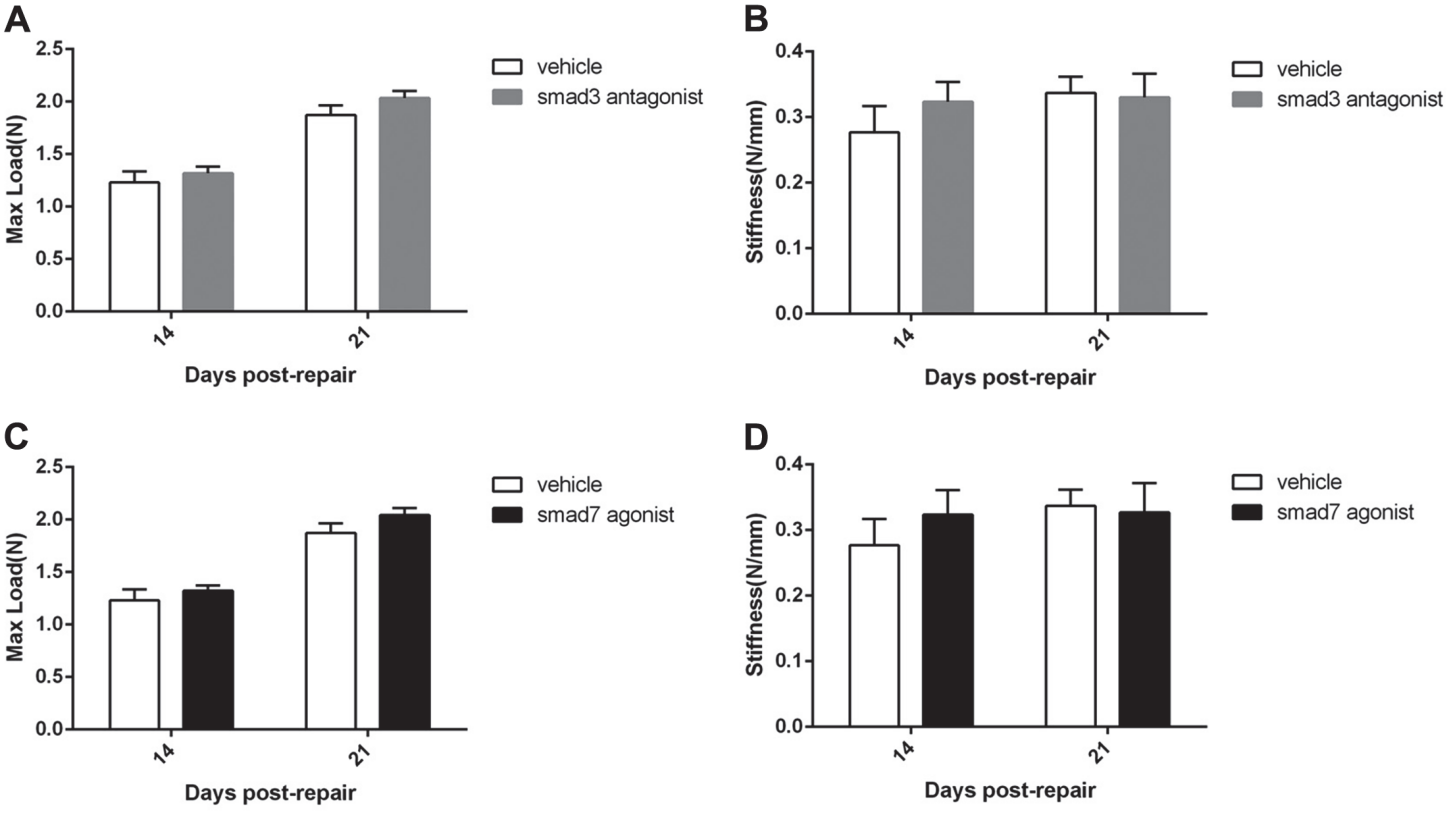
Determined the max load during flexor tendon healing at failure and stiffness on days 14 and 21 postrepair. (**A**) Effect of SMAD3 antagonist treatment on max load. (**B**) Effect of SMAD3 antagonist treatment on stiffness. (**C**) Effect of SMAD7 agonist treatment on max load. (**D**) Effect of SMAD7 agonist treatment on stiffness.

The maximum tensile load and the stiffness of the tendon tissues during flexor tendon healing were determined to evaluate the effect of the SMAD3 antagonist and SMAD7 agonist on the change of biomechanical properties after repair. No matter whether on day 14 or day 21, the maximum load in the e SMAD3 antagonist group and SMAD7 agonist group were higher than that in the corresponding control group, although there was no significant difference (Figs. 3A and 3C). In addition, the stiffness of the SMAD3 antagonist group and SMAD7 agonist group on day 14 were higher than that of their corresponding control group, although there was no significant difference, until day 21 the stiffness was comparable to that of their corresponding control group.(Figs. 3B and 3D).

### SMAD3 Antagonist and SMAD7 Agonist Improved the Pathological Changes during Flexor Tendon Healing

**Fig. 4:**
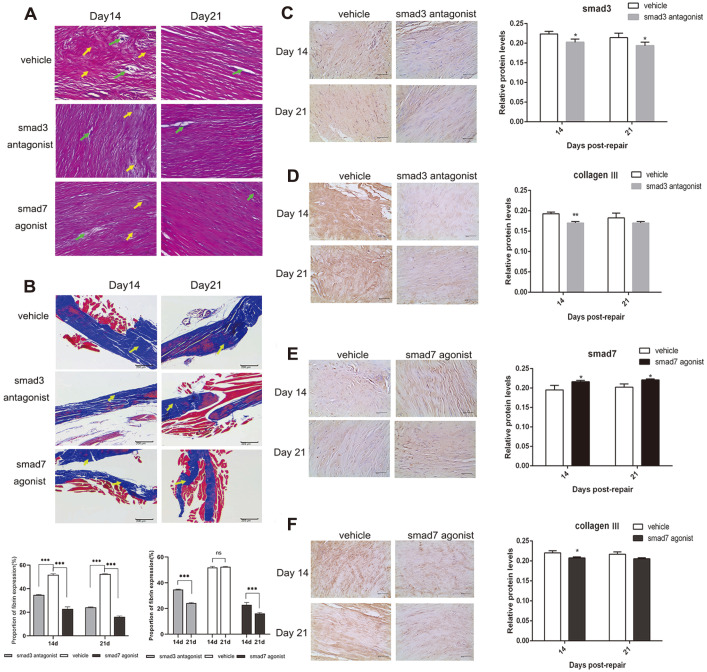
SMAD3 antagonist and SMAD7 agonist improved the pathological changes during flexor tendon healing. (**A**) HE staining of FDL tendon tissues on day 14 and day 21. Scale bars = 50 μm. B. Masson staining of FDL tendon tissues on day 14 and day 21 Scale bars = 200 μm. (**C-F**) Immunohistochemistry of SMAD3 (**C**), COLLAGEN III (**D** and **F**), and SMAD7 (**E**) were determined in FDL tendon tissues on day 14 and day 21. Scale bars = 40 μm. *p < 0.05, **p < 0.01, vs. vehicle group.

HE staining and Masson staining was used to observe the pathological changes of the repaired site on days 14 and 21. Pathological change of tendon tissue as Fig. 4A has shown, on day 14 post-repair, the disordered arrangement (yellow arrows) and remarkable necrosis (green arrows) of collagen fibers were observed in the vehicle group. By contrast, the SMAD3 antagonist group and SMAD7 agonist group showed a more orderly arrangement of collagen fibers, and less necrosis. On day 21 post-repair, compared with the vehicle group, the orderliness and tightness of collagen fibers were improving, and necrosis decreased too, in the SMAD3 antagonist group and SMAD7 agonist group. Moreover, the results of the Masson staining were consistent with it (Fig. 4B), while the yellow arrows point to fibrin. Using time as the variable, the longer the tendon recovery time, the fewer adhesions caused by fibrin. Using drugs as the variable, the application of SMAD3 antagonist or SMAD7 agonist developed fewer adhesions.

The effects of SMAD3 antagonist and SMAD7 agonist on COLLAGEN III protein expression in mouse FDL tendon tissues were analyzed by immunohistochemistry. The expressions of smad3 (Fig. 4C) and COLLAGEN III (Fig. 4D) protein in the SMAD3 antagonist group were significantly lower than that in the vehicle group on day 14 and day 21. The expression of COLLAGEN III in the SMAD3 antagonist group was significantly lower than that in the vehicle group on day 14, and there was no significant difference in the expression of COLLAGEN III between the SMAD3 antagonist group and the vehicle group on day 21 (Fig. 4D). In addition, significantly increased expression of SMAD7 was observed in the SMAD7 agonist group on both day 14 and day 21 (Fig. 4E). The expression of COLLAGEN III in the SMAD7 agonist group was significantly lower than that in the vehicle group on day 14, and there was no significant difference in the expression of COLLAGEN III on day 21 (Fig. 4F).

### SMAD3 Antagonist and SMAD7 Agonist Altered the Expressions of *Mmp2*, *Mmp9*, and *Scx* during Flexor Tendon Healing

**Fig. 5:**
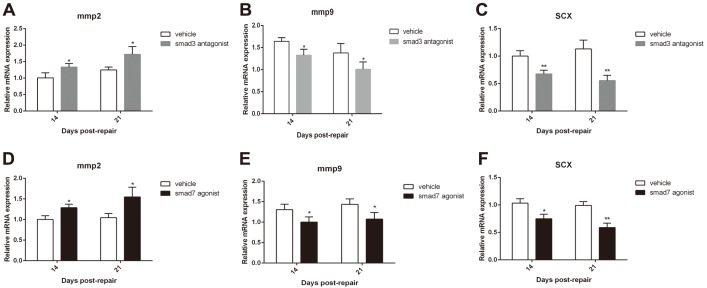
SMAD3 antagonist and SMAD7 agonist altered the expressions of *Mmp2*, *Mmp9*, and *Scx* after repair. (**A-F**) Relative mRNA expressions of *Mmp2* (**A** and **D**), *Mmp9* (**B** and **E**), and SCX (**C** and **F**) in FDL tendon tissues were determined by RT-qPCR. Expression was normalized to β-actin. *p < 0.05, **p < 0.01 vs. vehicle group.

The expression of *Mmp2*, *Mmp9*, and *Scx* was analyzed by RT-qPCR on day 14 and day 21 post-repair. The results showed that the expression levels of *Mmp2* in the SMAD3 antagonist group and SMAD7 agonist group were significantly higher than that in their corresponding vehicle group on days 14 and 21 (Figs. 5A and 5D), while the expression levels of *Mmp9* and *Scx* were significantly lower in the SMAD3 antagonist group and SMAD7 agonist group both than in their corresponding vehicle group on day 14 and 21 (Figs. 5B, 5C, 5E, and 5F).

## Discussion

Tendon adhesion seriously affects hand function and is an urgent problem to be solved in the field of hand surgery. The improvement of clinical efficacy depends on the continuous and in-depth study of its mechanism [2]. At present, most studies mainly prevent tendon adhesion by regulating the expression of cytokines and inhibiting the proliferation of tissue fibrocytes, and they are all in the research stage [25, 26]. Among many cytokines, TGF-β is recognized as the most closely related to adhesion formation [27, 28]. In the TGF-β/SMAD signal pathway, TGF-β realizes its function through the smad family, such as SMAD3 or SMAD7 [11, 18]. In addition, SMAD7 prevents SMAD3 phosphorylation, which ultimately damages the TGF-β1 signaling pathway. However, due to the absence of p-SMAD3, the downstream pathway cannot be activated [17]. This was consistent with our results. Our results showed that SMAD7 exerts its function by inhibiting the phosphorylation of SMAD3. Besides, either systemic inhibition of SMAD3 or activation of SMAD7 can reduce matrix deposition around the repair site, thereby reducing the formation of fibrous adhesions during FDL healing.

There are three types of smad family: receptor-activated SMAD (R-SMAD), co-mediated SMAD (co-SMAD), and inhibitory SMAD (I-SMAD) [29]. Among them, SMAD3 belongs to R-SMAD and mediates TGF-β1 signaling. SMAD7 belongs to I-SMAD, which has a negative feedback effect on TGF-β1/SMAD signaling pathway [30]. In this study, *Smad3* was up-regulated on day 7 and significantly increased on day 14, while *Smad7* was down-regulated on day 7 and significantly decreased on day 14. Suggesting that there may be an accelerated healing process of flexor tendon injury from days 7 to 14 and that *Smad3* and *Smad7* had a sustained peak from days 14 to 21. The functional consequence of adhesion formation was increasing the range of motion (ROM) on the finger. Here, total ROM and sliding resistance, a measure of the overall work of bending, were assessed by in-situ tests [23]. Therefore, on the 7th and 14th days, we verified these results by applying a SMAD3 antagonist and SMAD7 agonist. Our results showed that both the SMAD3 antagonist and SMAD7 agonist reduced sliding resistance and increased the range of motion of the joint. In the process of tendon healing, the content imbalance of the smad family is one of the main causes of fibrosis [14], thus rebalancing SMAD7/SMAD3 signaling is critical during the remodeling phase of tendon repair. These biomechanical results suggested that SMAD3 inhibition or SMAD7 activation can effectively reduce adhesion formation during flexor tendon healing. Moreover, SMAD7 inhibited the phosphorylation of SMAD3 in the healing process, suggesting that rebalancing SMAD7/SMAD3 signaling plays an important role in the formation of flexor tendon adhesion.

The repair site of tendon injury initially bristled with granulation tissue dominated by type III COLLAGEN [31]. Therefore, the expression of collagen III plays an important role in catabolic and anabolic responses to a tendon injury. Previous studies had shown that TGF-β1 inhibition resulted in reduced adhesion after tendon repair [32, 33]. We detected a corresponding decrease in type III COLLAGEN production after smad3 inhibition or smad7 activation, and immunohistochemistry confirmed a parallel decrease in protein expression from day 14 to day 21 after repair. This implied that SMAD3 or SMAD7 were involved in the regulation of collagen formation and tissue remodeling during tendon healing, that was, SMAD3 antagonism or SMAD7 excitation could inhibit adhesion. The decrease of matrix deposition during tendon repair and reconstruction was associated with the decrease in type III collagen production, which was consistent with the reports that SMAD3 dysfunction inhibited type III COLLAGEN [34].

Scleraxis (*Scx*) gene was the best characterization marker of tendon morphogenesis which encode a basic helix-loop-helix (bHLH) transcription factor, and there was some evidence that Scx activation can induce tendon regeneration [35], which plays a significant role in tendon development and maturation [36]. Dyment *et al*. [37] have found that SCX is not expressed in paratendinous tissues (mainly digital tendon sheaths) under normal conditions, but increased after tendon injury in a mouse. In this study, we found that the expression of *Scx* was lower than the vehicle group on days 14 and 21 in SMAD3 antagonism or SMAD7 agonism mice. This might be one of the reasons for the reduction of adhesions, which also meant SMAD3 inhibition or SMAD7 activation reduced the expression of Scx, consistent with Lorda-Diez's study [38].

Early tendon healing is associated with molecular events of cellular inflammation, and histologic examination showed an increase in inflammatory cells number following surgical resection of the flexor tendon between 7 and 14 days after surgery [39]. Changes in the MMP family during tendon healing were another important factor in the formation of adhesive texture, studies have shown that when tendonitis occurs, the activity of MMP2 and MMP9 would change [40]. Farhat *et al*. found that MMP2 degradation leads to excessive deposition of extracellular matrix and type III COLLAGEN, and promotes the development of tendon adhesion [41]. Loiselle *et al*. also found that the degree of tendon adhesion in *Mmp9* knockout mice was significantly reduced [21]. While MMP2 and MMP9 are gelatinases mainly involved in the destruction of damaged collagen [40, 42]. In the study, tendon repairs in smad3 antagonism or smad7 agonism mice had a signiﬁcant increase in *Mmp2* expression and a significant decrease in *Mmp9* expression. These findings turned out that SMAD3 and SMAD7 may regulate the degradation of COLLAGEN by these matrix metalloproteinases to control adhesion formation, thereby reducing scar formation. Thus, SMAD3 antagonism or SMAD7 agonism improved adhesion during tendon healing by modifying extracellular matrix components. All results suggest that SMAD7 inhibited flexor tendon adhesion by blocking of SMAD3 phosphorylation.

This study provided references for the regulation of SMAD7 and SMAD3 in the adhesion formation of flexor tendon healing, and provided a theoretical basis for the SMAD7-SMAD3 signal cascade during flexor tendon adhesion healing. This signal complemented the regulatory mechanisms associated with the TGF-β/SMADs signal in the adhesion formation of flexor tendon, which may represent a therapeutic approach for flexor tendon adhesion. Nevertheless, there were some limitations we wanted to address. This model was used to study zone II injuries, and we did not use true zone II tear injuries. In addition, although it is important to explore the relative expression of different genes, expression alone does not represent the whole picture of translation, activity, and gene metabolism.

In summary, adhesion formation after flexor tendon injury healing might be regulated by the SMAD7-SMAD3 signaling cascade in the TGF-β/SMAD pathway, which provided a possible theoretical basis for adhesion inhibition during flexor tendon healing.

## Figures and Tables

**Table 1:** Primers of genes for RT-qPCR.

Gene name	following primers 5'-3'	Reverse primers 5'-3'
*Mmp2*	TCAGTCGATCACTAGCGTCAAT	CTAACTTCTCCCCAC AGGGA
*Mmp9*	GCCGTCTACTCCTCCCCGTGT	GTCTCTCTCCTACCCTCTGG
*Smad3*	GCAGGCTCTCCA AACCTCT	GTGGAATGTCTCCCCAACTC
*Smad7*	CTCAAACCAACTGAGACTGTC	AGGCTCCAGAAGAAGTTGGG
*Scx*	GACCTAAAGAGGCGGCATGA	ACCTCTCCATCTGTCTCCCC
*β-actin*	GAAGATCAAGATCATTGCTCC	TACTCCTGCTTGCTGATCCA

## References

[1] Tang JB (2005). Clinical outcomes associated with flexor tendon repair. Hand Clin..

[2] Karakaplan M, Kilinc O, Ceylan MF, Ertem K, Aslanturk O (2021). Mid-term results of two-stage tendon reconstruction of zone II flexor tendon injuries. Niger. J. Clin. Pract..

[3] Peters SE, Jha B, Ross M (2021). Rehabilitation following surgery for flexor tendon injuries of the hand. Cochrane Database Syst. Rev..

[4] Liu C, Yu K, Bai J, Tian D (2018). Experimental study of tendon sheath repair via decellularized amnion to prevent tendon adhesion. PLoS One.

[5] Kvist M, Józsa L, Järvinen MJ, Kvist H (1987). Chronic *Achilles paratenonitis* in athletes: a histological and histochemical study. Pathology.

[6] Mailey B, O'Shea G, Romanelli M, West B (2021). Systemic immunosuppression for prevention of recurrent tendon adhesions. Plast. Reconstr. Surg. Glob. Open.

[7] Harrison R, Mudera V, Grobbelaar A, Jones M, McGrouther D (2003). Synovial sheath cell migratory response to flexor tendon injury: an experimental study in rats. J. Hand Surg. Am..

[8] Maggi R, Maggi C (2002). Tendon surgery in Brown's syndrome. J. Pediatr. Ophthalmol. Strabismus.

[9] Rowlands DS, Shultz SP, Ogawa T, Aoi W, Korte M (2014). The effects of uniquely-processed titanium on biological systems: implications for human health and performance. J. Funct. Biomater..

[10] Wu LM, Wang JK, Liu J, Fan CC, Wang YJ, Xiong Y (2021). Gait analysis combined with the expression of TGF-β1, TGF-β3, and CREB during Achilles tendon healing in rat. Chin. J. Traumatol..

[11] Wang D, Pun CCM, Huang S, Tang TCM, Ho KKW, Rothrauff BB (2020). Tendon-derived extracellular matrix induces mesenchymal stem cell tenogenesis via an integrin/transforming growth factor-β crosstalk-mediated mechanism. FASEB J..

[12] Wu C, Jiang J, Boye A, Jiang Y, Yang Y (2014). Compound *Astragalus* and *Salvia miltiorrhiza* extract suppresses rabbits' hypertrophic scar by modulating the TGF-β/Smad signal. Dermatology.

[13] Zhang K, Fang T, Shao Y, Wu Y (2021). TGF-β-MTA1-SMAD7-SMAD3-SOX4-EZH2 signaling axis promotes viability, migration, invasion and EMT of hepatocellular carcinoma cells. Cancer Manag. Res..

[14] Yang Q, Chen HY, Wang JN, Han HQ, Jiang L, Wu WF (2020). Alcohol promotes renal fibrosis by activating Nox2/4-mediated DNA methylation of Smad7. Clin. Sci..

[15] Nagar H, Kim S, Lee I, Kim S, Choi SJ, Piao S (2021). Downregulation of CR6-interacting factor 1 suppresses keloid fibroblast growth via the TGF-β/Smad signaling pathway. Sci. Rep..

[16] Flanders KC, Major CD, Arabshahi A, Aburime EE, Okada MH, Fujii M (2003). Interference with transforming growth factorbeta/Smad3 signaling results in accelerated healing of wounds in previously irradiated skin. Am. J. Pathol..

[17] Zhong C, Zhang YF, Huang JH, Wang ZY, Chen QY, Su LT (2017). The Chinese medicine, Jianpi Huayu Decoction, inhibits the epithelial mesenchymal transition via the regulation of the Smad3/Smad7 cascade. Am. J. Transl. Res..

[18] Jiang K, Chun G, Wang Z, Du Q, Wang A, Xiong Y (2016). Effect of transforming growth factor-β3 on the expression of Smad3 and Smad7 in tenocytes. Mol. Med. Rep..

[19] Loiselle AE, Bragdon GA, Jacobson JA, Hasslund S, Cortes ZE, Schwarz EM (2009). Remodeling of murine intrasynovial tendon adhesions following injury: MMP and neotendon gene expression. J. Orthop. Res..

[20] Jiang K, Li Y, Xiang C, Xiong Y, Jia J (2021). TGF-β3 regulates adhesion formation through the JNK/c-Jun pathway during flexor tendon healing. BMC Musculoskelet. Disord..

[21] Loiselle AE, Frisch BJ, Wolenski M, Jacobson JA, Calvi LM, Schwarz EM (2012). Bone marrow-derived matrix metalloproteinase-9 is associated with fibrous adhesion formation after murine flexor tendon injury. PLoS One.

[22] Hasslund S, Jacobson JA, Dadali T, Basile P, Ulrich-Vinther M, Søballe K (2008). Adhesions in a murine flexor tendon graft model: autograft versus allograft reconstruction. J. Orthop. Res..

[23] (2010). Adhesions in a murine flexor tendon graft model: Autograft versus allograft reconstruction. J. Orthop. Res..

[24] Loiselle A, Bragdon G, Jacobson J, Hasslund S, Cortes Z, Schwarz E (2009). Remodeling of murine intrasynovial tendon adhesions following injury: MMP and neotendon gene expression. J. Orthop. Res..

[25] Ackerman JE, Best KT, O'Keefe RJ, Loiselle AE (2017). Deletion of EP4 in S100a4-lineage cells reduces scar tissue formation during early but not later stages of tendon healing. Sci. Rep..

[26] Jelinsky SA, Li L, Ellis D, Archambault J, Li J, St Andre M (2011). Treatment with rhBMP12 or rhBMP13 increase the rate and the quality of rat Achilles tendon repair. J. Orthop. Res..

[27] Kerwin L, El Tal A, Stiff M, Fakhouri T (2014). Scar prevention and remodeling: a review of the medical, surgical, topical and light treatment approaches. Int. J. Dermatol..

[28] Loiselle A, Yukata K, Geary M, Kondabolu S, Shi S, Jonason J (2015). Development of antisense oligonucleotide (ASO) technology against Tgf-β signaling to prevent scarring during flexor tendon repair. J. Orthop. Res..

[29] Derynck R, Zhang Y (2003). Smad-dependent and Smad-independent pathways in TGF-beta family signalling. Nature.

[30] Moustakas A, Souchelnytskyi S, Heldin C (2001). Smad regulation in TGF-beta signal transduction. J. Cell Sci..

[31] Beredjiklian P (2003). Biologic aspects of flexor tendon laceration and repair. J. Bone Joint Surg. Am..

[32] Bates S, Morrow E, Zhang A, Pham H, Longaker M, Chang J (2006). Mannose-6-phosphate, an inhibitor of transforming growth factor-beta, improves range of motion after flexor tendon repair. J. Bone Joint Surg. Am..

[33] Chang J, Thunder R, Most D, Longaker M, Lineaweaver W (2000). Studies in flexor tendon wound healing: neutralizing antibody to TGF-beta1 increases postoperative range of motion. Plast. Reconstr. Surg..

[34] Katzel E, Wolenski M, Loiselle A, Basile P, Flick L, Langstein H (2011). Impact of Smad3 loss of function on scarring and adhesion formation during tendon healing. J. Orthop. Res..

[35] Murchison ND, Price BA, Conner DA, Keene DR, Olson EN, Tabin CJ (2007). Regulation of tendon differentiation by scleraxis distinguishes force-transmitting tendons from muscle-anchoring tendons. Development.

[36] Sakabe T, Sakai K, Maeda T, Sunaga A, Furuta N, Schweitzer R (2018). Transcription factor scleraxis vitally contributes to progenitor lineage direction in wound healing of adult tendon in mice. J. Biol. Chem..

[37] Wang X, Chen H, Liu W, Liu M, Zhou D, Chen Q (2020). The association of plasma high-density lipoprotein cholesterol levels and cirrhosis development in obese patients with chronic hepatitis B: a cohort study. Eur. J. Gastroenterol. Hepatol..

[38] Lorda-Diez C, Montero J, Martinez-Cue C, Garcia-Porrero J, Hurle JJTJobc (2009). Transforming growth factors beta coordinate cartilage and tendon differentiation in the developing limb mesenchyme. J. Biol. Chem..

[39] Ding B, Wang X (2019). Photochemical tissue bonding technique for improving healing of hand tendon injury. Surg. Innov..

[40] Oshiro W, Lou J, Xing X, Tu Y, Manske P (2003). Flexor tendon healing in the rat: a histologic and gene expression study. J. Hand Surg..

[41] Islam SS, Mokhtari RB, El Hout Y, Azadi MA, Alauddin M, Yeger H (2014). TGF-β1 induces EMT reprogramming of porcine bladder urothelial cells into collagen producing fibroblasts-like cells in a Smad2/Smad3-dependent manner. J. Cell Commun. Signal..

[42] Bramono D, Richmond J, Weitzel P, Kaplan D, Altman G (2004). Matrix metalloproteinases and their clinical applications in orthopaedics. Clin. Orthop. Relat. Res..

